# How much incisor decompensation is achieved prior to orthognathic surgery?

**DOI:** 10.4317/jced.51310

**Published:** 2014-07-01

**Authors:** Calum McNeil, Grant T. McIntyre, Sean Laverick

**Affiliations:** 1 Undergraduate student. Orthodontic Department, Dundee Dental Hospital, 2 Park Place, Dundee, DD1 4HR, UK; 2 Consultant, Senior Lecturer. Orthodontic Department, Dundee Dental Hospital, 2 Park Place, Dundee, DD1 4HR, UK; 3 Consultant. Oral and Maxillofacial Surgery Department, Ninewells Hospital, Dundee, DD1 9SY, UK

## Abstract

Objectives: To quantify incisor decompensation in preparation for orthognathic surgery. Study design: Pre-treatment and pre-surgery lateral cephalograms for 86 patients who had combined orthodontic and orthognathic treatment were digitised using OPAL 2.1 [http://www.opalimage.co.uk]. To assess intra-observer reproducibility, 25 images were re-digitised one month later. Random and systematic error were assessed using the Dahlberg formula and a two-sample t-test, respectively. Differences in the proportions of cases where the maxillary (1100 +/- 60) or mandibular (900 +/- 60) incisors were fully decomensated were assessed using a Chi-square test (p<0.05). Mann-Whitney U tests were used to identify if there were any differences in the amount of net decompensation for maxillary and mandibular incisors between the Class II combined and Class III groups (p<0.05). Results: Random and systematic error were less than 0.5 degrees and p<0.05, respectively. A greater proportion of cases had decompensated mandibular incisors (80%) than maxillary incisors (62%) and this difference was statistically significant (p=0.029). The amount of maxillary incisor decompensation in the Class II and Class III groups did not statistically differ (p=0.45) whereas the mandibular incisors in the Class III group underwent statistically significantly greater decompensation (p=0.02). Conclusions: Mandibular incisors were decompensated for a greater proportion of cases than maxillary incisors in preparation for orthognathic surgery. There was no difference in the amount of maxillary incisor decompensation between Class II and Class III cases. There was a greater net decompensation for mandibular incisors in Class III cases when compared to Class II cases.

** Key words:**Decompensation, orthognathic, pre-surgical orthodontics, surgical-orthodontic.

## Introduction

Approximately 4% of the population have dentofacial deformity requiring combined surgical-orthodontic treatment (1) and these patients present with a variable amount of dentoalveolar compensation. This occurs in all three planes of space; anteroposterior, vertical and lateral but is most evident in the anteroposterior plane.

Pre-surgical orthodontic treatment consists of three concurrent aspects: arch alignment, arch co-ordination and arch decompensation (2). In most centers, incisor decompensation is achieved with fixed appliances, whereby the incisors are either proclined or retroclined so that the incisors are at the correct axial inclination to the maxillary or mandibular skeletal bases (3). The outcome of the pre-surgical orthodontic phase influences the magnitude of the movements that can be achieved at the time of surgery (4) as the occlusion is used as a surgical template, in addition to the information derived from the lateral cephalomteric prediction planning (5). Adequate decompensation also facilitates the possibility of fully corrected inter-arch relationships at the time of surgery by optimising the surgical movements. Thus the desired facial and occlusal changes are provided with adequate pre-surgical incisor decompensation, which also minimises the need for protracted post-surgical orthodontic treatment. As a result, incisor decompensation is one of the major contributing factors towards the overall aesthetic and functional outcome (1) and without adequate incisor decompensation the surgical change and the final skeletal position may limited by the extent of the incisor overjet (6). Consequently, decompensation also has implications for long-term stability (7).

Capelozza Filho *et al.* (4) found decompensation was more successful in the mandibular arch than in the maxillary arch in Class III surgical patients. Furthermore, where the maxillary teeth were adequately decompensated, this resulted in greater surgical correction. Capelozza Filho *et al.* (4) also found that there was a strong correlation between decompensation and postsurgical mandibular excess / lower anterior face height in their Class II group. Johnston *et al.* (1) examined a UK sample of subjects with Class III malocclusion, and found that although most patients achieved a normal overjet with surgery, only 40% of the sample had a normal ANB angle, and 52% had an excessive SNB angle after treatment. Although they did not investigate decompensation, they attributed the cases where surgical skeletal correction was limited to be due to inadequate decompensation. Xu *et al.* (8) found that in cases of mandibular hyperplasia, the incisors could be decompensated to an equivalent level of a group of similar non-surgical cases.

Troy *et al.* (9) similarly found only a 50% improvement in the upper incisor inclination when they were proclined at the pre-treatment stage in class III patients, whilst the majority of the mandibular arches were satisfactorily decompensated before surgery. Furthermore, Ahn and Baek (10) found decompensation was adequate in the mandibular arch for between 23.53% and 100% of the patients they studied. Interestingly, mandibular incisor decompensation has been shown to be incomplete in 28% of patients being treated for a severe Class II malocclusion and more successful where there was greater pretreatment mandibular incisor proclination (11). In a Chinese sample of patients treated for Class III malocclusion, Xu and Qin (12) noted that maxillary premolar extractions enhanced maxillary incisor decompensation, resulting in an improvement in mandibular position after surgery.

However, none of these investigations determined the delivery of incisor decompensation in a complete cohort of patients of all malocclusion groups scheduled for orthognathic surgery in a state-funded healthcare system. This is an area that therefore requires further investigation.

The aims of this study were to determine if maxillary and mandibular incisors are adequately decompensated in preparation for orthognathic surgery and to quantify any differences between the maxillary and mandibular incisors for patients presenting with Class II and Class III malocclusion

## Material and Methods

One hundred consecutive patients who underwent maxillary and/or mandibular orthognathic surgery at a university dental hospital from 1 January 2005 onwards were included. Within the cohort, the following cases were excluded ( Table 1).

**Table 1:**
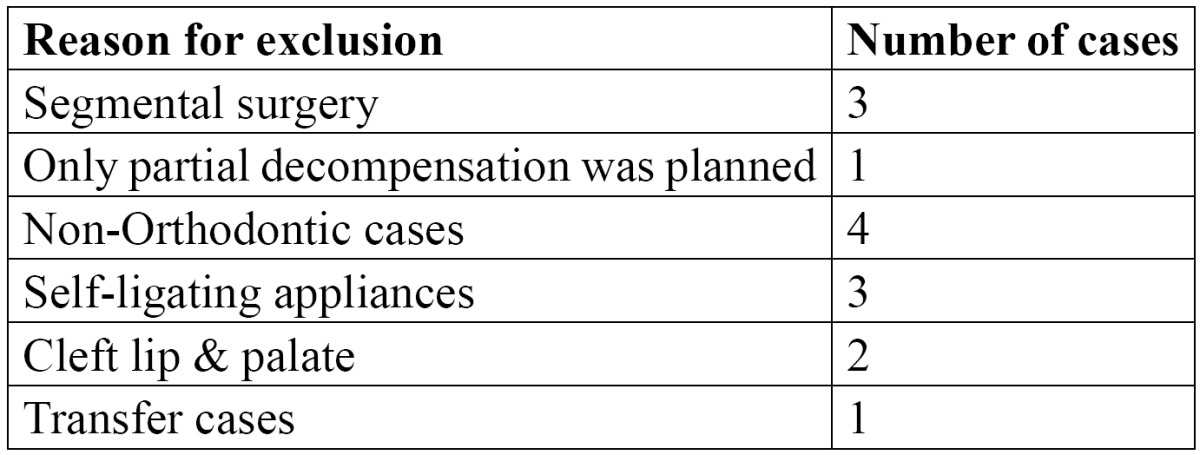
Exclusion of cases.

All the cases were treated by, or under the direction of a Consultant Orthodontist with the 0.022 inch-slot MBT prescription appliance [3M-Unitek, Monrovia, California, USA]. The ‘surgical’ archwire was an 0.019x0.025 inch stainless steel archwire. Where necessary, elastics and auxiliary archwires were used, particularly to retrocline / procline the maxillary / mandibular incisors in Class II and Class III cases as necessary (2).

The pre-treatment and pre-surgery lateral cephalograms were recorded before the start of orthodontic treatment and at the end of pre-surgical orthodontic treatment, respectively. These were digitised using OPAL 2.1 lateral cephalometric system [British Orthodontic Society, London] [http://www.opalimage.co.uk]. This was installed on a Lenovo R61 machine attached to a Lenovo 2-button USB optical mouse and 15.4-inch TFT active matrix monitor with 1280x800 resolution, aspect ratio 16:10, pixel pitch 0.2373 and contrast ratio 628:1 [www.lenovo.com]. Data were extracted and analysed using Microsoft Excel [Redmond, California] to determine whether the incisors were decompensated to the normal range for a Caucasian population: 1100 +/- 60 and 900 +/- 60 for the maxillary and mandibular incisors, respectively (13). Values were rounded up or down to the nearest 0.1 degree in relation to the pixel pitch value of the monitor.

## Statistical Analysis

To assess intra-observer reproducibility, 25 lateral cephalograms were digitised on two separate occasions one month apart by the same operator using the same technique in accordance with Houston (14). Random error was assessed using the Dahlberg formula (15) and systematic error was calculated using a two-sample t-test (14). The level of significance was *p* < 0.05 for the systematic error (14).

Descriptive statistics were used to summarise the whole sample. A Chi-square test was used to determine if there was a statistically significant difference in the proportion of cases where the maxillary or mandibular incisors were fully decompensated [*p*<0.05]. The data were categorized into Class II (11,16) and Class III incisor groups along with upper and lower incisor groups. Mann-Whitney *U* tests were used to determine if there were any statistically significant differences between Class II combined (16) / Class III cases for maxillary and mandibular incisor decompensation [*p*<0.05].

## Results

Random error and systematic error were both less than 0.5 degrees and *p*<0.05, respectively.

The pre-treatment and/or pre-surgery lateral cephalometric radiographs were missing for two patients leaving 86 patients. Of these, 4 presented with a Class I malocclusion [underlying skeletal open bite], 23 with a Class II division 1 malocclusion, 7 with a Class II division 2 malocclusion and 52 with a Class III malocclusion.

Sixty-three percent of the maxillary incisor group were judged to be adequately decompensated whilst the value for the mandibular incisors was 80% ( Table 2). This difference was statistically significant [p=0.029].

**Table 2:**
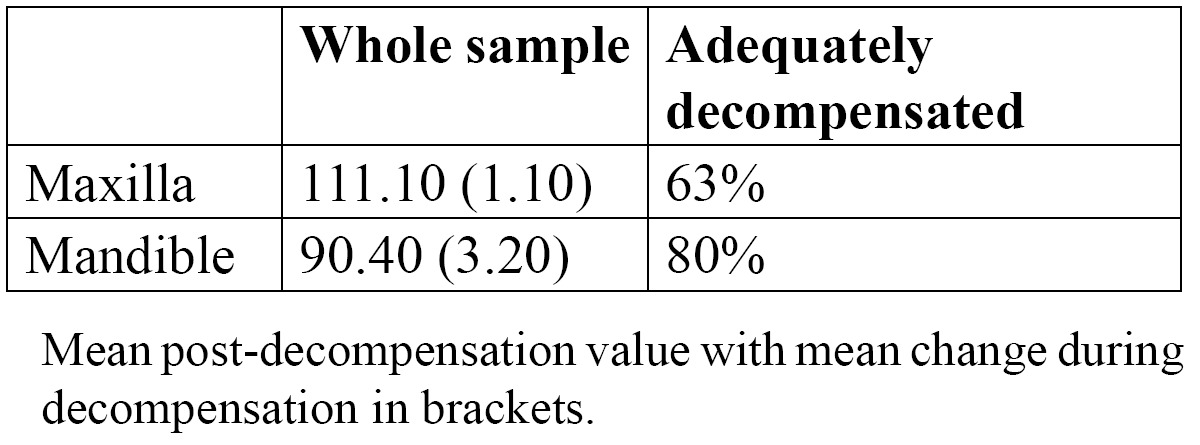
Decompensation values and proportion of fully decompensated maxillary and mandibular incisors.

The amount of maxillary incisor decompensation in the Class II malocclusion and Class III malocclusion groups did not statistically differ [*p*=0.45] but the amount of tooth movement for the mandibular incisors was statistically significantly greater [*p*=0.02] in the Class III group than in the Class II group ( Table 3).

**Table 3:**
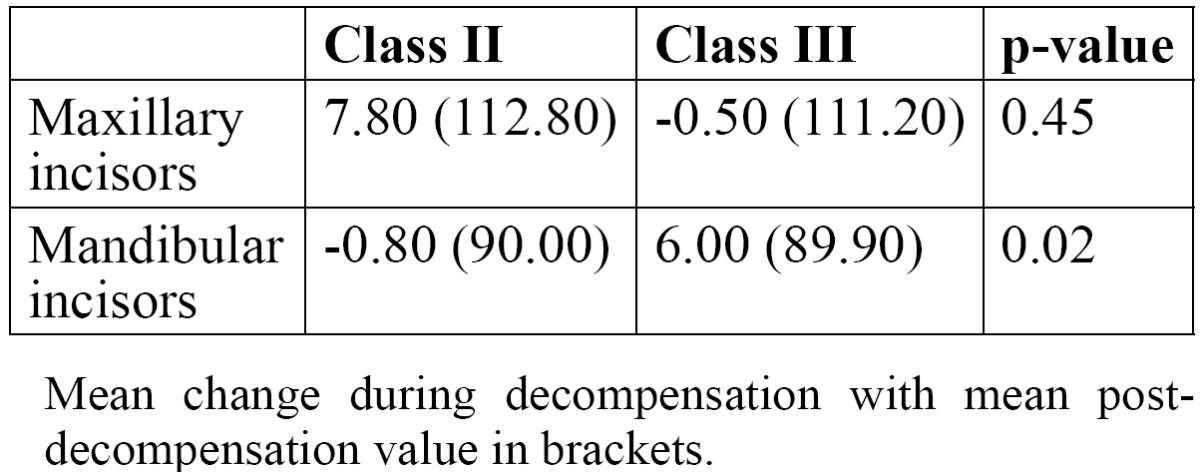
Incisor changes for Class II and Class III cases.

## Discussion

We found that adequate decompensation was more likely to be achieved in the mandible [80%] than in the maxilla [63%] and this difference was statistically significant. That not all patients were fully decompensated before surgery is in line with other investigations. Ahn and Baek (10) found lower incisor decompensation was adequate for between 23.53% and 100% of patients whilst Troy *et al.* (9) found only a 50% improvement in the upper incisor inclination when they were proclined at the pre-treatment stage. Troy *et al.* (9) however, only studied class III patients whereas we included all incisor relationship groups. Other studies paradoxically suggest that it is more difficult to decompensate mandibular incisors adequately (1,5,6). This is an area that therefore requires further investigation.

This appears to be the first study that has investigated the differences between the maxillary and mandibular incisors and between patients presenting with either a Class II or Class III malocclusion. We found no statistically significant difference for the mean change during decompensation for maxillary incisors between the Class III and Class II group, whilst the amount of decompensation achieved for the mandibular incisors for Class III patients was statistically significantly greater than in the Class II group. Interestingly, Potts *et al.* (5) found that most cases with retroclined incisors were not decompensated adequately prior to orthognathic surgery, whilst Ari-Demirkaya and Ilhan (17) identified that in 28% of patients with Class II malocclusion, the mandibular incisors were still protrusive at the time of surgery with angles greater than 99o. Proclination of incisors has been shown to be more achievable than retroclination (6) and our results would indicate that incisor decomensation in the mandible is more achievable than in the maxilla. Our value of 20% of the mandibular incisors remaining compensated at surgery is in line with these findings and is an improvement on the value of 28% for patients with Class II malocclusion as reported by Burden *et al.* (11). However, we also included Class I and Class III patients in addition to Class II patients. It should be noted that the results from the UK national study of Class III patients treated by Consultant Orthodontists further support the results in this study where almost half of the patients still had retroclined lower incisors and around a third still had proclined upper incisors at the end of pre-surgical orthodontic treatment (1).

There are a number of possible explanations for full decompensation not being achieved when desired. In Class III cases, inadequate labial bone and lack of periodontal support to allow sufficient advancement of incisors, previous mandibular arch extractions, lower lip neuromuscular resistance to mandibular incisor advancement and poor patient compliance with intra-oral elastic traction are all possible reasons (1) whilst in cases of mild crowding, the decision to not extract teeth can negatively affect the amount of decompensation that can be achieved (18).

The cohort was completely ascertained as all surgical-orthodontic treatment in this region is undertaken by the NHS. Cases where full decompensation was not planned were excluded as the data for these cases would introduce bias. However, one limitation of this study was the lack of completeness of the records due to the conventional film radiographs for two patients being lost. When analyzing subgroups, the numbers of subjects can become small and in this study there were only seven patients with a class II division 2 incisor relationship. The Class II division 1 and division 2 cases were combined for analysis as per Burden *et al.* (11) and Proffit *et al.* (16). There were variables that were not considered in this study which could have influenced the results such as extractions, the severity of the initial skeletal discrepancy and the amount of bodily incisor movement required during pre-surgical decompensation. These were beyond the scope of a cephalometric investigation.

The results of this study have implications for clinical practice. Clinicians should be aware of the need to fully decompensate incisors [where clinically appropriate] in advance of orthognathic surgery. As this has been shown to be more difficult in the maxilla, careful attention should be paid to pre-surgical orthodontic biomechanics. Fixed appliances are used along with inter-proximal reduction, extractions, molar distalization [where appropriate] (3) and corticotomy (19,20). With the advent of self-ligating appliances, the benefit of secure and robust archwire engagement offers a potential advantage for achieving a greater degree of decompensation, however any benefit may be lost due to the greater manufacturing tolerances between archwires and brackets in these systems (21). Therefore a future study should investigate any differences between conventional ligation and self-ligating appliances to assess if this variable has any effect on the level of decompensation. Interestingly, Kim *et al.* (22) found that pre-surgical orthodontic treatment involves extrusion of the incisors and premolars in addition to mandibular incisor proclination and in some cases arch expansion. A future study should also investigate the relative contribution of each type of movement to the overall arch dimensions at the time of surgery.

## Conclusions

Mandibular incisors were decompensated for a greater proportion of cases than maxillary incisors in preparation for orthognathic surgery.

There was no difference in the amount of maxillary incisor decompensation between Class II and Class III cases.

There was a greater net decompensation for mandibular incisors in Class III cases when compared to Class II cases.

## References

[1] Johnston C, Burden D, Kennedy D, Harradine N, Stevenson M (2006). Class III surgical-orthodontic treatment: a cephalometric study. Am J Orthod Dentofac Orthop.

[2] Jacobs JD, Sinclair PM (1983). Principles of orthodontic mechanics in orthogathic surgery cases. Am J Orthod.

[3] Carlos VB, Giovanni O, Diego R, Angela S, Baccetti T (2009). Orthodontic decompensation in class III patients by means of distalization of upper molars. Prog Orthod.

[4] Capelozza Filho L, Martins A, Mazzotini R, da Silva Filho OG (1996). Effects of dental decomensation on the surgical treatment of mandibular prognathism. In J Adult Orthod Orthognath Surg.

[5] Potts B, Shanker S, Fields HW, Vig KW, Beck FM (2009). Dental and skeletal changes associated with Class II surgical-orthodontic treatment. Am J Orthod Dentofac Orthop.

[6] Potts B, Fields HW, Shanker S, Vig KW, Beck FM (2011). Dental and skeletal outcomes for Class II surgical-orthodontic treatment: A comparison between novice and experienced clinicians. Am J Orthod Dentofac Orthop.

[7] Lim LY, Cunningham SJ, Hunt NP (1998). Stability of mandibular incisor decompensation in orthognathic patients. In J Adult Orthod Orthognath Surg.

[8] Xu B, Hagg U, Tideman H, Piette E (1995). Presurgical orthodontic decompensation of mandibular incisors. Aust Orthodo J.

[9] Troy BA, Shanker S, Fields HW, Vig K, Johnston W (2009). Comparison of incisor inclination in patients with Class III malocclusion treated with orthognathic surgery or orthodontic camouflage. Am J Orthod Dentofac Orthop.

[10] Ahn HW, Baek SH (2011). Skeletal anteroposterior discrepancy and vertical type effects on lower incisor preoperative decompensation and postoperative compensation in skeletal Class III patients. Angle Orthod.

[11] Burden D, Johnston C, Kennedy D, Harradine N, Stevenson M (2007). A cephalometric study of Class II malocclusions treated with mandibular surgery. Am J Orthod Dentofac Orthop.

[12] Xu B, Qin K (2012). The effect of extraction and non-extraction decompensation to bimaxillary orthognathic surgery in skeletal class III malocclusion. Hua Xi Kou Qiang Yi Xue Za Zhi.

[13] Tan SS, Ahmad S, Moles DR, Cunningham SJ (2011). Picture archiving and communications systems: a study of reliability of orthodontic cephalometric analysis. Eur J Orthod.

[14] Houston WJ (1983). The analysis of errors in orthodontic measurements. Am J Orthod.

[15] Springate SG (2012). The effect of sample size and bias on the reliability of estimates of error: a comparative study of Dahlberg's formula. Eur J Orthod.

[16] Proffit WR, Phillips C, Douvartzidis N (1992). A comparison of outcomes of orthodontic and surgical orthodontic treatment of Class II malocclusion in adults. Am J Orthod Dentofac Orthop.

[17] Ari-Demirkaya A, Ilhan I (2008). Effects of relapse forces on periodontal status of mandibular incisors following orthognathic surgery. J Periodontol.

[18] Phonpraserth A, Cunningham SJ, Hunt NP (1999). Soft tissue changes associated with incisor decompensation prior to orthognathic surgery. Int J Adult Orthod Orthognath Surg.

[19] Kim SH, Kim I, Jeong DM, Chung KR, Zadeh H (2011). Corticotomy-assisted decompensation for augmentation of the mandibular anterior ridge. Am J Orthod Dentofacial Orthop.

[20] Ahn HW, Lee DY, Park YG, Kim SH, Chung KR, Nelson G (2012). Accelerated decompensation of mandibular incisors in surgical skeletal class III patients by using augmented corticotomy: a preliminary study. Am J Orthod Dentofacial Orthop.

[21] Bhalla NB, Good SA, McDonald F, Sherriff M, Cash AC (2010). Assessment of slot sizes in self-ligating brackets using electron microscopy. Aust Orthod J.

[22] Kim YI, Choi YK, Park SB, Son WS, Kim SS (2012). Three-dimensional analysis of dental decompensation for skeletal Class III malocclusion on the basis of vertical skeletal patterns obtained using cone-beam computed tomography. Korean J Orthod.

